# Sharp Object in the Belly: A Case of Pediatric Intentional Razor Blade Ingestion in the Emergency Department

**DOI:** 10.7759/cureus.7699

**Published:** 2020-04-16

**Authors:** Brandon M Carius, P. M Dodge, Brit Long

**Affiliations:** 1 Emergency Medicine, Brian D. Allgood Army Community Hospital, Camp Humphreys, KOR; 2 Prehospital Medicine, University of New Hampshire, Manchester, USA; 3 Emergency Medicine, Brooke Army Medical Center, Fort Sam Houston, USA

**Keywords:** foreign object, ingestion, child, endoscopy

## Abstract

Ingested foreign object (FOB) is a common complaint in the emergency department (ED), especially in pediatric patients. While many FOB ingestions are benign, sharp objects, including razor blades, are of particular concern given the acute risk of perforation throughout the gastrointestinal tracts. The majority of razor blade ingestions involve prisoners and psychiatric patients, which can complicate the diagnosis and treatment. Although literature suggests that risks of perforation and complication may be high, limited research available on sharp FOB ingestions supports a general non-interventional strategy. Instead, close follow-up and serial radiographs for natural passage are recommended for the majority of cases. We highlight the case of a 17-year-old female who presented to the ED for suspected FOB ingestion and was found to have a singular 3.0 x 0.5 cm razor blade on abdominal radiograph following an unremarkable initial evaluation. In line with prior literature, surgical consult supported natural passage with serial radiographs, and the patient was subsequently discharged home with a recommended bulk food diet.

## Introduction

Suspected foreign object (FOB) ingestion can cause significant concern in the emergency department (ED), especially when surgical intervention may be needed in 36% of cases [1]. A sharp FOB increases risk of perforation by 35%, further increasing provider and patient angst [2]. Here, we describe the case of intentional ingestion of a razor blade in a pediatric patient and its subsequent non-operative management.

## Case presentation

**Figure 1:**
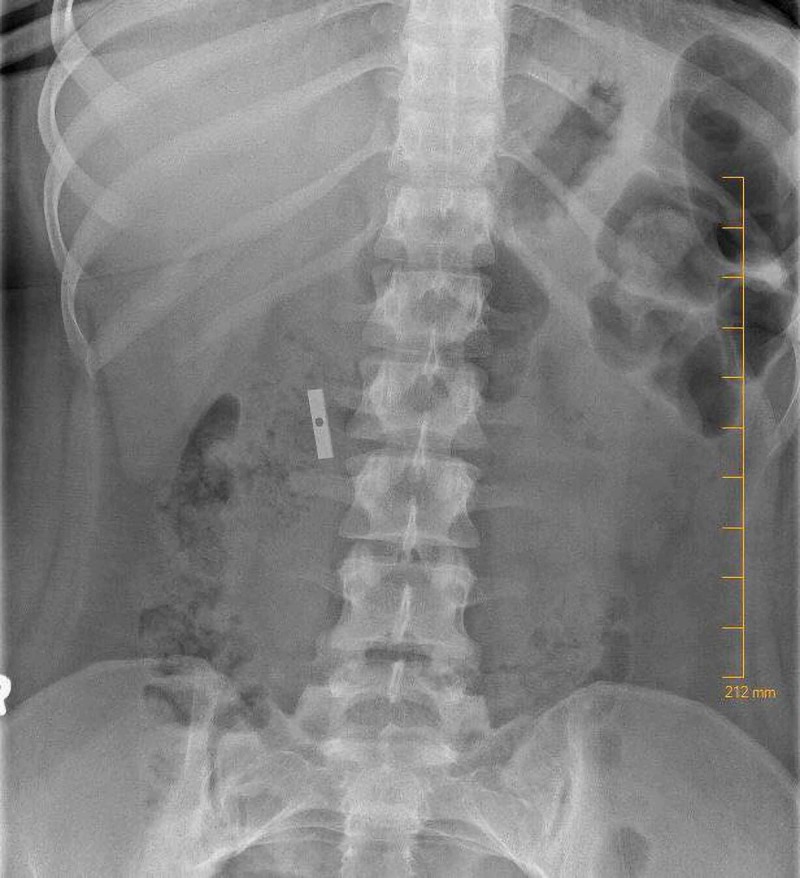
Anteroposterior radiograph demonstrates an isolated metallic object in the right upper quadrant consistent with suspected razor blade ingestion.

**Figure 2:**
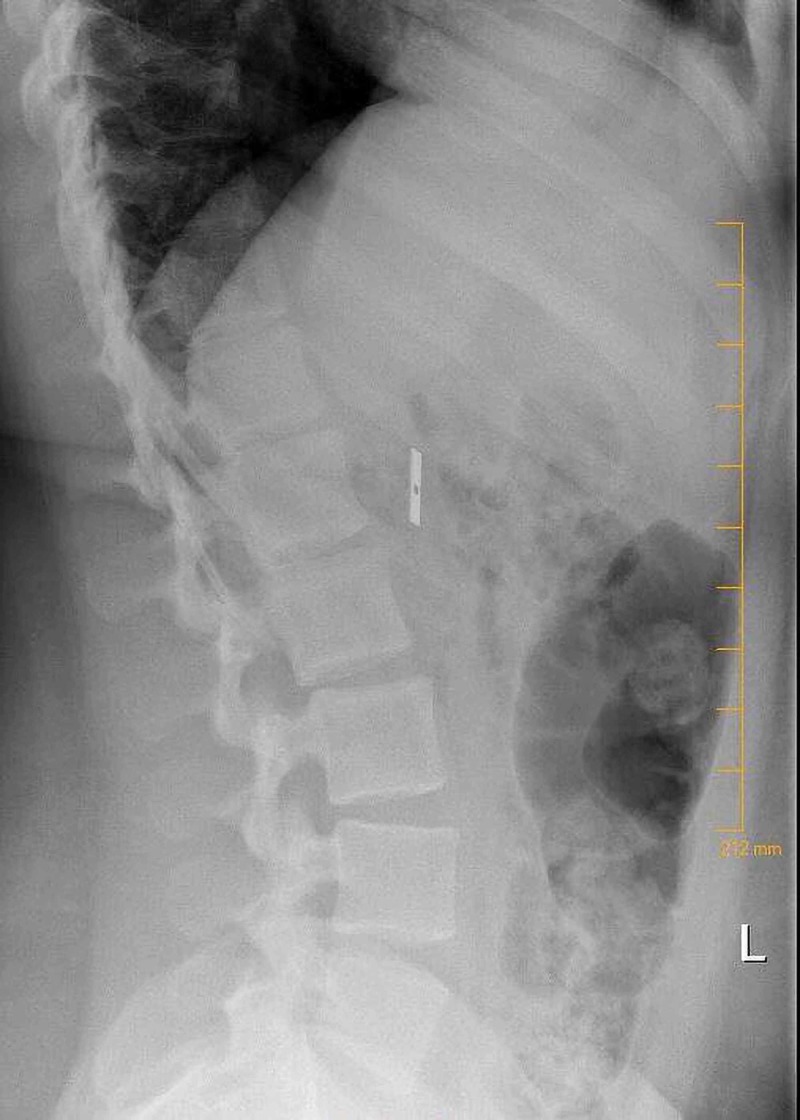
Lateral radiograph demonstrates an isolated metallic object in the abdomen consistent with suspected razor blade ingestion.

A 17-year-old female with a prior history of FOB ingestion presented with her parents who were concerned for a new ingestion. Upon questioning, the patient admitted to disassembling a pencil sharpener and swallowing the blade approximately 24 hours prior to presentation. She denied throat, chest, or abdominal pain; rectal bleeding; or melena. The patient’s blood pressure, heart rate, and temperature were normal. Physical examination was unremarkable, including no abdominal tenderness or guarding. No fecal occult blood or anemia was noted on laboratory testing. A radiograph of the kidneys, ureter, and bladder revealed a 3.0 x 0.5 cm rectangular radiopaque foreign body located within the right upper quadrant near the hepatic flexure, consistent with the stated history (Figures 1, 2). Consults were obtained and agreed the object was most likely in the colon, and therefore the best course was to allow for natural passage with a bulk foods diet and close follow-up. The patient was lost to follow-up, but she did not return to the ED for further care.

## Discussion

Overviews on FOB ingestion are numerous, but the topic of an ingested sharp FOB, especially in the pediatric patient, is less frequently discussed, and definitive positions on management are rare. Generally FOB ingestions reveal a wide variety of intentional and non-intentional causes, but case reports and series of sharp FOBs in adults demonstrate a near-universal presentation of patients who are prisoners and/or have significant psychiatric comorbidities [1-6]. Symptoms most commonly include abdominal pain and vomiting, but many cases also demonstrate anorexia and constipation on presentation [1,2,7]. Patients often wrap the razor blades in gum or paper, which is theorized to make ingestions less a commitment to do serious self-injury, and more a deliberate action to gain attention and care with underlying psychiatric illness [2,4,7].

Initial suspicion of sharp FOB ingestion like razor blades is almost entirely based upon history or witnessed consumption. Prior literature has not demonstrated any consistent examination findings in suspected ingestions, although cases of perforation may reveal mediastinal emphysema and/or peritoneal signs with significant pain, tenderness, and a rigid abdomen depending on the FOB location [4,5]. Diagnosis for razor blade ingestion is typically made on plain film radiographs, as the metallic material is radiopaque and can be reasonably correlated with the position in the gastrointestinal tract, although one case series utilized computed tomography for more precise localization [8]. Additional evaluation can include hemoglobin and hematocrit counts, as well as fecal occult blood testing, if bleeding is suspected. One report found inconsistent elevations of C-reactive protein and white blood cell counts, but this is not demonstrated elsewhere in literature [1].

The general consensus in literature is for a wait-and-see approach to allow for passage of FOBs, but sharp objects warrant increased concern. Most reviews and reports, including the most recent American Society of Gastrointestinal Endoscopy management guidelines, quote a nearly 100-year-old figure of increased perforation rates with sharp FOBs as high as 35% [1,9]. This number is consistent with a Spanish case series of 167 patients that found a perforation rate of 33% with sharp FOBs and a series of 11 prisoners in Ireland which demonstrated a need for surgical intervention in 36% of sharp FOB ingestion [1,10]. The specific concern for sharp FOB ingestions is primarily within the initial stages of passage. A site of considerable narrowing with strong peristalsis, the lower esophagus is commonly cited as the primary site of perforation and often considered as reason for immediate retrieval regardless of patient presentation [1,10-12]. Other areas of concern are those with significant angulation or stricture including the ileocecal junction, the hepatic and splenic flexures, and the rectosigmoid junction. The gastric pylorus and ileocecal valve will not accommodate FOBs greater than 2 cm in diameter [5]. Additionally, chronic conditions causing scarring and stricture, such as ulcerative colitis, could make transit more difficult and increase the chance of injury.

There are no specific recommendations for razor blades or other sharp FOB ingestions in the pediatric population. General guidelines advocate conservative management once sharp FOBs pass the proximal duodenum [10,13]. For FOBs beyond this point, close follow-up is recommended with daily plain films to document passage and emergent return precautions for pain, rectal bleeding, and melena [1,10]. If the object does not progress in three days, surgical intervention should then be considered [1,5,10,13]. Otherwise, supportive care centers on a bulk food diet with high-fiber foods like fruits, vegetables, and whole grains. Strict return precautions should be given for acute worsening of symptoms to include pain, nausea, vomiting, and bloody emesis or stool, at which point emergent laparotomy should be considered [5,10].

## Conclusions

In this case of intentional razor blade ingestion in a pediatric patient, no further return visits to the ED were made in the four weeks following initial presentation. Uncomplicated passage of swallowed sharp FOB is consistent with the majority of cases found in literature, although concerns for obstruction, perforation, or other visceral injury are warranted and require strict return precautions to hemodynamically stable patients with unremarkable evaluations.
